# Survival nomogram for high-grade bladder cancer patients after surgery based on the SEER database and external validation cohort

**DOI:** 10.3389/fonc.2023.1164401

**Published:** 2023-06-16

**Authors:** Yihe Li, Tao Chen, Bin Fu, Yixing Luo, Luyao Chen

**Affiliations:** ^1^ Department of Urology, The First Affiliated Hospital of Nanchang University, Nanchang, China; ^2^ Department of Gastroenterology, The First Affiliated Hospital of Nanchang University, Nanchang, China

**Keywords:** high-grade bladder cancer, SEER database, prognosis, nomogram, radical cystectomy

## Abstract

**Background:**

The aim of this study was to develop a comprehensive and effective nomogram for predicting overall survival (OS) rates in postoperative patients with high-grade bladder urothelial carcinoma.

**Methods:**

Patients diagnosed with high-grade urothelial carcinoma of the bladder after radical cystectomy (RC) between 2004 and 2015 in the Surveillance, Epidemiology, and End Results (SEER) database were enrolled. We randomly split (7:3) these patients into the primary cohort and the internal validation cohort. Two hundred eighteen patients from the First Affiliated Hospital of Nanchang University were collected as the external validation cohort. Univariate and multivariate Cox regression analyses were carried out to seek prognostic factors of postoperative patients with high-grade bladder cancer (HGBC). According to these significant prognostic factors, a simple-to-use nomogram was established for predicting OS. Their performances were evaluated using the concordance index (C-index), the receiver operating characteristic (ROC) curves, calibration curves, and decision curve analysis (DCA).

**Results:**

The study included 4,541 patients. Multivariate Cox regression analysis demonstrated that T stage, positive lymph nodes (PLNs), age, chemotherapy, regional lymph nodes examined (RLNE), and tumor size were correlated with OS. The C-index of the nomogram in the training cohort, internal validation cohort, and external validation cohort were 0.700, 0.717, and 0.681, respectively. In the training, internal validation, and external validation cohorts, the ROC curves showed that the 1-, 3-, and 5-year areas under the curve (AUCs) were higher than 0.700, indicating that the nomogram had good reliability and accuracy. The results of calibration and DCA showed good concordance and clinical applicability.

**Conclusion:**

A nomogram was developed for the first time to predict personalized 1-, 3-, and 5-year OS in HGBC patients after RC. The internal and external validation confirmed the excellent discrimination and calibration ability of the nomogram. The nomogram can help clinicians design personalized treatment strategies and assist with clinical decisions.

## Introduction

Bladder cancer (BLCA) is the most common urinary tract malignancy worldwide, and its incidence rate is increasing gradually. According to global cancer statistics, an estimated 81,180 new BLCA cases and almost 17,100 BLCA deaths occurred in the United States in 2022 (1). Although age-standardized morbidity and mortality are declining over the past 20 years, the number of BLCA events is increasing globally, and the burden of BLCA is likely to rise in the future due to trends in population aging and environmental pollution (2, 3). Ninety-five percent of BLCA cases are urothelial carcinomas, including forms of divergent differentiation (4). Urothelial cancer is a highly diverse disease, ranging from low-grade tumors that are less threatening to high-grade tumors that can be fatal (5, 6). The grade of urothelial carcinoma is particularly important in non-invasive diseases, especially papillary tumors, which are closely associated with the recurrence and invasive behavior of BLCA. High-grade bladder cancer (HGBC) is aggressive with a high risk of recurrence, progression, and metastasis and a poor prognosis, whereas low-grade bladder cancer (LGBC) has a relatively good prognosis (7). Radical cystectomy (RC) and pelvic lymph node dissection (PLND) are usually recommended for high-grade non-muscle-invasive bladder cancer (NMIBC) with a high risk of tumor progression and muscle-invasive bladder cancer (MIBC) (4, 8, 9).

In a Canadian multi-medical center follow-up study, 33% of patients undergoing RC recurred and the 5-year overall survival (OS) rate was <60% (10). Therefore, there is a need for separate analyses to identify the prognostic factors most associated with OS in HGBC patients after RC and to individualize the management of postoperative patients to improve the effectiveness of surgical treatment. However, the development of predictive models for OS in HGBC patients after RC has not been performed so far.

Nomograms serve as useful statistical models to integrate relevant factors to predict individual tumor prognosis, advancing the development of personalized medicine and assisting physicians in developing treatment plans (11). In our study, we obtained the clinical and pathological characteristics of HGBC patients undergoing RC from 2004 to 2015 from the Surveillance, Epidemiology, and End Results (SEER) database and identified the risk factors to build a practical nomogram that helps predict overall survival time. In addition, we evaluated the performance of the nomogram and validated the applicability of the nomogram internally and externally separately to make the prediction model more convincing.

## Materials and methods

### Patient cohorts

**Figure 1:**
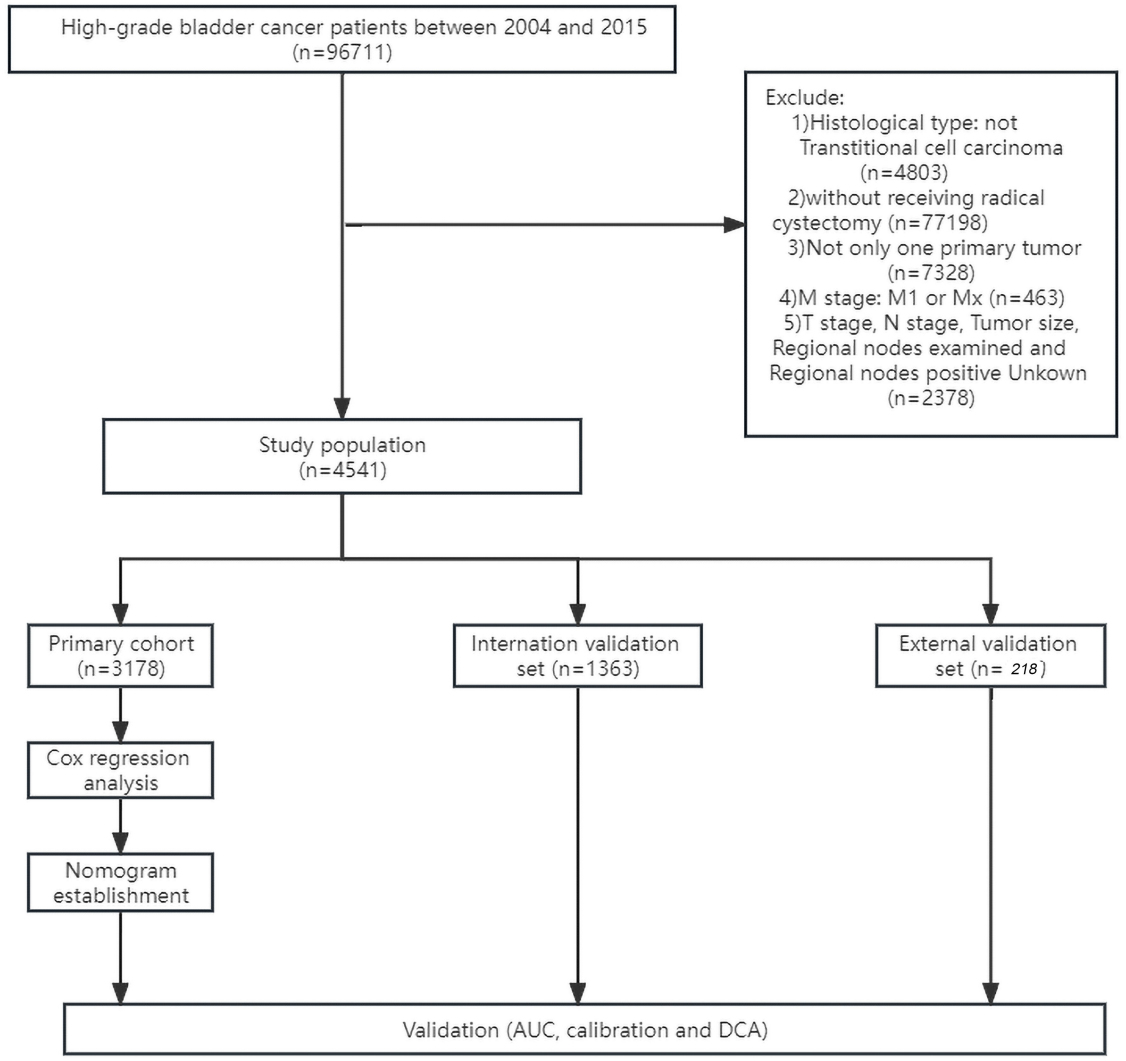
Flowchart for screening patient records from the SEER database.

The SEER*Stat software (version 8.3.6) was applied to extracted data including demographic and clinical characteristics. In our study, HGBC patients in the SEER database registered from 2004 to 2015 were selected. Patients enrolled in our study met the following inclusion criteria: high-grade BLCA patients diagnosed from 2004 to 2015. The exclusion criteria were as follows: a) histological type: not urothelial carcinoma; b) did not receive radical cystectomy; c) not only one primary tumor; d) M stage: M1 or Mx; and e) T stage, N stage, tumor size, regional lymph nodes examined, and regional nodes positive unknown. **Figure 1** shows the detailed screening procedure. Because SEER is a public database and all records are de-identified, no additional ethical approval or informed consent is required to access the data after signing the SEER Research Data Agreement.

The external validation cohort was composed of 218 BLCA patients receiving RC at the First Affiliated Hospital of Nanchang University between 2010 and 2021. The inclusion and exclusion criteria were in accordance with the SEER database. The last follow-up time was December 2022. Our study was approved by the Ethics Committee of the First Affiliated Hospital of Nanchang University.

### Nomogram construction and statistical analyses

The baseline characteristics of the included population were depicted in numbers and percentages. Univariate and multivariate Cox regression models were applied to calculate odds ratios (ORs) and 95% confidence intervals (CIs) to determine independent risk variables related to OS. Then, we used the connected risk factors to establish a simple-to-use nomogram. The calibration and discrimination of the model were assessed by the C-index, receiver operating characteristic (ROC) curve, the area under the curve (AUC) (12), and calibration plots (13). In addition, we further evaluated the clinical utility by decision curve analysis (DCA) (14). All statistical analysis was completed by using SPSS (version 24.0; SPSS, Armonk, NY, USA, Inc.) and R software (version 4.2.0). We considered a two-tailed *p*-value <0.05 as statistically significant.

## Results

### Baseline characteristics

Based on the screening criteria, we enrolled a total of 4,541 high-grade BLCA patients receiving RC from the SEER database, which consisted of 3,178 patients in the primary cohort and 1,363 patients in the internal validation cohort. **Table 1** shows the demographic and pathological characteristics of the patients. There was no significant difference in demographic information and clinical features between the primary cohort and the internal validation cohort. Most patients were between 60 and 80 years old (64.2%) and men (67.5%). Most tumors were T2/3 (75.3%) and N0 (69.3%). Tumors >40 mm in size (47.7%) were more common. The number of RLNE was frequently <10 (36.1%), and the number of PLN was mostly 0 (70.1%). Less than half of the patients received chemotherapy (45.8%), and most patients did not receive radiotherapy (97.1%). In the external validation cohort from our medical center, most patients were between 60 and 80 years old (71.6%) and men (86.2%). More than half of the tumors were T3 (46.8%) and N0 (66.1%). The tumor size between 20 and 40 mm (55.5%) was more common. The number of RLNE was frequently between 11 and 20 (47.7%), and the number of PLN was usually 0 (68.3%). A minority of the patients received chemotherapy (23.9%), and most patients did not receive radiotherapy (95.9%). The clinical data of the patients from our medical center are listed in **Table 2**.

**Table 1:** Characteristics of high-grade BLCA patients from the SEER database.

Variables	All patients (%) 4,541	Primary cohort (%) 3,178	Validation cohort (%) 1,363	*p*
Age (years)				0.921
<60	1,235 (27.2)	860 (27.1)	375 (27.5)	
60–80	2,917 (64.2)	2,044 (64.3)	873 (64.0)	
>80	389 (8.6)	274 (8.6)	115 (8.4)	
Sex				0.881
Male	3,064 (67.5)	2,147 (67.6)	917 (67.3)	
Female	1,477 (32.5)	1,031 (32.4)	446 (32.7)	
T stage				0.187
T1/Tis/Ta	378 (8.3)	252 (7.9)	126 (9.2)	
T2	1,662 (36.6)	1,147 (36.1)	515 (37.8)	
T3	1,758 (38.7)	1,258 (39.6)	500 (36.7)	
T4	743 (16.4)	521 (16.4)	222 (16.3)	
N stage				0.354
N0	3,146 (69.3)	2,191 (68.9)	955 (70.1)	
N1	697 (15.3)	485 (15.3)	212 (15.6)	
N2	676 (14.9)	489 (15.4)	187 (13.7)	
N3	22 (0.5)	13 (0.4)	9 (0.7)	
Tumor size				0.163
<20 mm	554 (12.2)	393 (12.4)	161 (11.8)	
20–40 mm	1,823 (40.1)	1,247 (392)	576 (42.3)	
>40 mm	2,164 (47.7)	1,538 (48.4)	626 (45.9)	
Regional nodes examined				0.469
<10	1,640 (36.1)	1,166 (36.7)	474 (34.8)	
10–20	1,459 (32.1)	1,012 (31.8)	447 (32.8)	
>20	1,442 (31.8)	1,000 (31.5)	442 (32.4)	
Positive lymph nodes				0.615
0	3,185 (70.1)	2,219 (69.8)	966 (70.9)	
1–5	1,140 (25.1)	802 (25.2)	338 (24.8)	
>5	216 (4.8)	157 (4.9)	59 (4.3)	
Chemotherapy				0.923
No	2,461 (54.2)	1,722 (54.2)	739 (54.2)	
Yes	2,080 (45.8)	1,456 (45.8)	624 (45.8)	
Radiotherapy				0.819
No	4,410 (97.1)	3,088 (97.2)	1,322 (97.30)	
Yes	131 (2.9)	90 (2.8)	41 (3.0)	

**Table 2:** Clinicopathological characteristics of high-grade BLCA patients in the SEER and Chinese cohorts.

Variables	SEER cohort (%) 4,541	Chinese cohort (%) 218	*p*
Age (years)			0.078
<60	1,235 (27.2)	49 (22.5)	
60–80	2,917 (64.2)	156 (71.6)	
>80	389 (8.6)	13 (6.0)	
Sex			<0.001
Male	3,064 (67.5)	188 (86.2)	
Female	1,477 (32.5)	30 (13.8)	
T stage			<0.001
T1/Tis/Ta	378 (8.3)	30 (13.8)	
T2	1,662 (36.6)	37 (17.0)	
T3	1,758 (38.7)	102 (46.8)	
T4	743 (16.4)	49 (22.5)	
N stage			0.011
N0	3,146 (69.3)	144 (66.1)	
N1	697 (15.3)	32 (14.7)	
N2	676 (14.9)	37 (17.0)	
N3	22 (0.5)	5 (2.3)	
Tumor size			<0.001
<20 mm	554 (12.2)	24 (11.0)	
20–40 mm	1,823 (40.1)	121 (55.5)	
>40 mm	2,164 (47.7)	73 (33.5)	
Regional nodes examined			<0.001
<10	1,640 (36.1)	43 (19.7)	
11–20	1,459 (32.1)	104 (47.7)	
>20	1,442 (31.8)	71 (32.6)	
Positive lymph nodes			0.029
0	3,185 (70.1)	149 (68.3)	
1–5	1,140 (25.1)	50 (22.9)	
>5	216 (4.8)	19 (8.7)	
Chemotherapy			<0.001
No	2,461 (54.2)	166 (76.1)	
Yes	2,080 (45.8)	52 (23.9)	
Radiotherapy			0.392
No	4,410 (97.1)	209 (95.9)	
Yes	131 (2.9)	9 (4.1)	

### Identification of prognostic predictors

Univariate and multivariate Cox regression analyses were performed to explore independent prognostic predictors of OS for HGBC patients after RC. Age, sex, T stage, N stage, tumor size, ELN, PLN, chemotherapy, and radiotherapy were included in the univariate Cox regression analysis. The results of the univariate Cox regression analysis revealed that age, T stage, N stage, tumor size, ELN, PLN, chemotherapy, and radiotherapy were identified as OS-related variables (*p* < 0.05) (**Table 3**), while sex had no significant difference (*p* > 0.05). Then, the multivariate Cox regression analysis was performed to eliminate confounding effects among OS-related variables (*p* < 0.05). Age, T stage, tumor size, ELN, PLN, and chemotherapy were identified as independent prognostic predictors of OS for high-grade BLCA patients (**Table 3**). Older age, higher T stage, larger tumor size, and more PLN were associated with poor prognosis. More RLNE and receiving chemotherapy were connected with better oncology outcomes.

**Table 3:** Univariate and multivariate Cox proportional hazards regression analyses in the training set.

	Univariate analysis			Multivariate analysis		
	OR	95% CI	*p*	OR	95% CI	*p*
Age (years)						
<60	Reference			Reference		
60–80	1.276	1.137–1.431	<0.001	1.247	1.111–1.340	<0.001
>80	2.450	1.631–2.311	<0.001	1.647	1.377–1.970	<0.001
Sex						
Male	Reference			Reference		
Female	1.121	1.013–1.241	0.027	1.066	0.962–1.180	0.223
T stage						
T1/Tis/Ta	Reference			Reference		
T2	1.418	1.101–1.827	0.007	1.447	1.122–1.868	0.004
T3	3.202	2.507–4.088	<0.001	2.639	2.056–3.386	<0.001
T4	4.594	3.560–5.928	<0.001	3.485	2.676–4.538	<0.001
N stage						
N0	Reference			Reference		
N1	2.157	1.904–2.442	<0.001	0.962	0.512–1.806	0.903
N2	2.954	2.615–3.336	<0.001	1.285	0.682–2.421	0.438
N3	5.325	3.191–8.885	<0.001	1.997	0.860–4.636	0.107
Tumor size						
<20 mm	Reference			Reference		
20–40 mm	1.234	1.044–1.458	0.0146	1.043	0.880–1.236	0.628
>40 mm	1.617	1.374–1.903	<0.001	1.268	1.073–1.499	0.005
ELN						
<10	Reference			Reference		
11–20	0.808	0.722–0.905	<0.001	0.829	0.740–0.929	<0.001
>20	0.682	0.605–0.768	<0.001	0.673	0.596–0.760	<0.001
PLN						
0	Reference			Reference		
1–5	2.448	2.207–2.716	<0.001	1.861	1.144–5.110	0.042
>5	3.676	3.059–4.418	<0.001	2.351	1.226–4.508	0.01
Chemotherapy						
No	Reference			Reference		
Yes	0.905	0.821–0.998	0.045	0.706	0.636–0.783	<0.001
Radiotherapy						
No	Reference			Reference		
Yes	1.597	1.241–2.055	<0.001	1.171	0.906–1.513	0.226

### Nomogram construction

**Figure 2:**
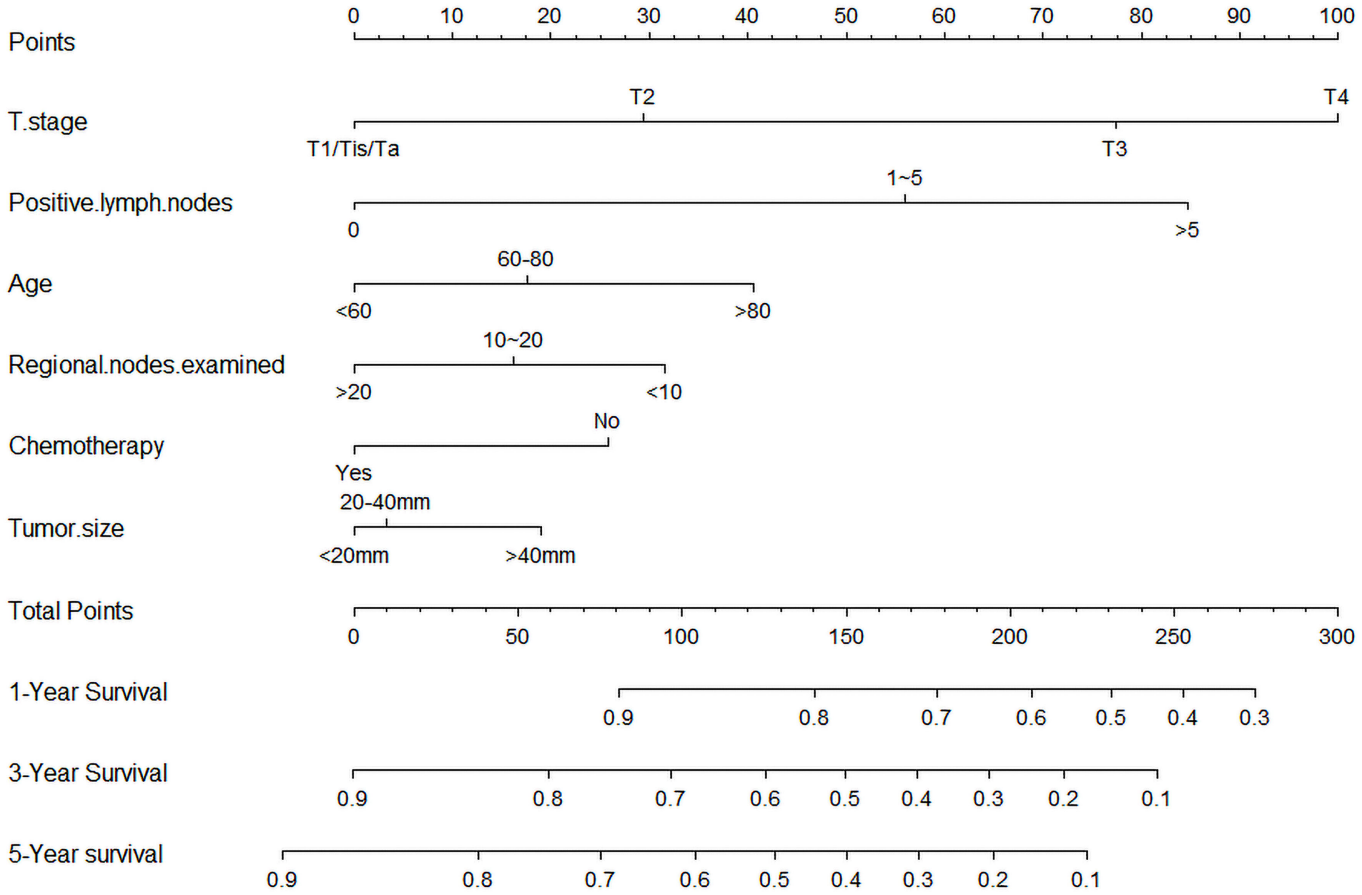
Nomogram predicting the 1-, 3-, and 5-year bladder cancer overall survival probabilities for high-grade bladder cancer patients after radical cystectomy. The variables include T stage, positive lymph nodes, age, chemotherapy, regional nodes examined, and tumor size.

To predict the OS of HGBC patients, we developed a nomogram based on all the above independent OS-related predictors from the multivariate Cox regression analysis (**Figure 2**). The nomogram also offered each independent prognostic predictor a point. Adding these points could predict the 1-, 3-, and 5-year OS of HGBC patients after RC. As shown in **Figure 2**, chemotherapy and more RLNE are protective predictors for HGBC patients. The poor prognosis of HGBC patients was associated with higher T stage, more PLN, older age, and larger tumor size.

### Performance of the nomogram

**Figure 3:**
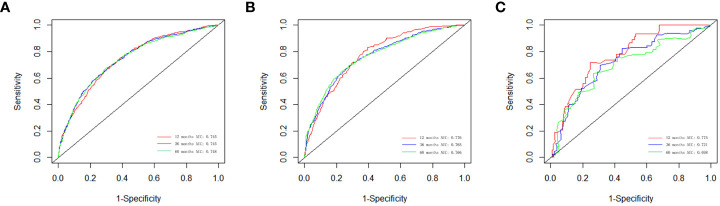
The ROC curves for the nomogram in the training cohort **(A)**, the internal validation cohort **(B)**, and the external validation cohort **(C)**.

**Figure 4:**
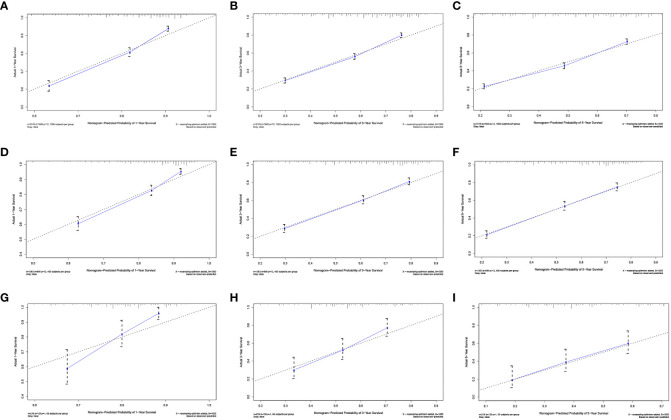
Calibration plots of the nomogram for 1 **(A)**, 3 **(B)**, and 5 years **(C)** in the development cohort; calibration plots of the nomogram for 1 **(D)**, 3 **(E)**, and 5 years **(F)** in the internal validation cohort; and calibration plots of the nomogram for 1 **(G)**, 3 **(H)**, and 5 years **(I)** in the internal validation cohort.

**Figure 5:**
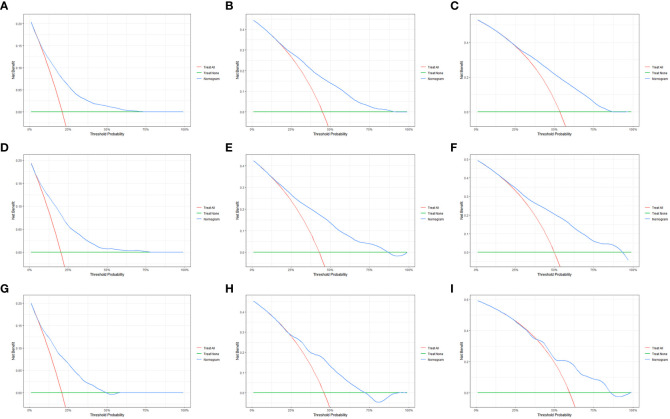
The decision curve analysis (DCA) of the OS nomogram at 1 **(A)**, 3 **(B)**, and 5 years **(C)** in the primary cohort; at 1 **(D)**, 3 **(E)**, and 5 years **(F)** in the internal validation cohort; and at 1 **(G)**, 3 **(H)**, and 5 years **(I)** in the external validation cohort.

We evaluated the performance of the nomogram using C-index, AUC, calibration curve, and DCA. The C-index of the nomogram in the training cohort, internal validation cohort, and external validation cohort was 0.700 (95% CI: 0.686–0.714), 0.717 (95% CI: 0.697–0.736), and 0.681 (95% CI: 0.636–0.726), respectively. Meanwhile, the ROC curve was applied to access the discriminative ability of the nomogram. The AUC values of the training cohort were 0.745, 0.745, and 0.748 for 1, 3, and 5 years, respectively; those of the internal validation cohort were 0.776, 0.765, and 0.766, respectively; and those of the external validation cohort were 0.775, 0.721, and 0.698, respectively (**Figure 3**). In addition, whether in the training, internal validation, or external validation cohort, the solid lines of the calibration curves of all cohorts were close to 45°, indicating that the predicted results were in good agreement with the actual observations (**Figure 4**). Furthermore, DCA showed that the nomogram had a high clinical application value and could be used as an effective auxiliary tool in clinical practice to maximize the benefit of postoperative patients (**Figure 5**).

## Discussion

BLCA is one of the major global health problems with a high morbidity and mortality rate. Although various treatments have been developed, RC with PLND remains the standard treatment method for MIBC and high-risk NMIBC. Based on the histological differences among cancer cells, bladder urothelial carcinoma was divided into high-grade and low-grade urothelial carcinoma of the bladder. Among high-risk NMIBC and MIBC, most of them are high-grade urothelial carcinoma, and high-grade bladder urothelial carcinoma has a high risk of recurrence and metastasis (7). Predictive models for OS for HGBC patients undergoing RC have never been available before. Therefore, it is necessary to explore the prognostic factors that affect the survival benefit of patients undergoing surgery in order to facilitate follow-up counseling and follow-up programs for HGBC patients undergoing RC.

Our study selected 4,541 HGBC patients from the SEER database, which were randomized to the development cohort (*n* = 3,178) and the validation cohort (*n* = 1,363) in a 7:3 ratio, and we also selected patients from our hospital (*n* = 218) as an external validation cohort. Based on the multivariate Cox regression analysis, T stage, positive lymph nodes, age, chemotherapy, RLNE, and primary tumor size were identified as independent prognostic predictors of OS. On the basis of these factors, we developed a nomogram to predict the OS of HGBC patients undergoing RC for 1, 3, and 5 years, and we further evaluated the performance of the nomogram. The findings suggest that T stage is the most important factor for the model. Several studies have shown that the depth of bladder infiltration significantly affects the prognosis of BLCA, as its aggressiveness and progressiveness increase significantly with the progression of T stage (15–18). The fact of more positive lymph nodes has been a disadvantage for patients undergoing RC, which is consistent with previous reports (8). Additionally, in this study, we found that the number of RLNE was significantly associated with OS of HGBC patients undergoing RC. With more RLNE, the prognosis is better (19–21). It suggested that lymph node dissection not only removes undetected micrometastases probably but also provides pathological information about regional lymph nodes to help clinicians develop subsequent treatment plans for this population (22). Age is also a prognostic factor, which may be related to the fact that elderly patients are more likely to have other chronic diseases. In the meantime, consistent with previous studies, chemotherapy helped improve OS (23, 24). In addition, our study also confirmed that primary tumor size was also associated with different oncology outcomes (25–27). Moreover, the results of the C-index and AUC proved that our nomogram had good concordance. In fact, the results of DCA confirm the clinical usefulness of our nomogram. In a word, the variables used to construct the prognostic model are statistically reliable in this study.

In recent years, many researchers have contributed to the development of postoperative predictive models for bladder cancer (26, 28–31). Although those models have been well validated externally, they may not be universally applicable. Because some of the incorporated variables are often difficult to obtain, such as lymphovascular infiltration, and are relatively complex to calculate, so further generalization of the prediction model is hindered. In addition, Yang et al. constructed a prognostic model to assess the cancer-specific survival of BLCA patients treated with RC (26). The final model included five variables: T stage, marital status, N stage, tumor size, and chemotherapy. However, the model lacked the factor of the number of RLNE and ignored the effect of individual PLND. Compared with previous prognostic models for BLCA patients treated with RC, we constructed an original predictive model for HGBC patients receiving RC.

However, this study has some limitations. First, our study was based on the SEER database and, therefore, lacked potentially important factors, such as preoperative laboratory results, lymphovascular infiltration, comorbidities, socioeconomic status, and Charlson comorbidity index, which can cause a significant impact on the prognosis of patients. Also, we excluded patients who have unknown information on many variables, which could lead to the appearance of selection bias. In addition, the SEER database lacks specific information on chemotherapy and surgical treatments (open or robot-assisted radical cystectomy, type of urinary diversion, and approach for urinary diversion). The factor of chemotherapy in our model represented that the patients have received chemotherapy, whether it is neoadjuvant or adjuvant chemotherapy. Moreover, the sequence of chemotherapy, the choice of chemotherapeutic drugs, and the surgical methods may have different prognostic effects (32–34). Previous strong evidence manifested that compared with open radical cystectomy, robot-assisted radical cystectomy had more advantages in reducing the blood transfusion rate by 50% and resulted in a statistically significant increase in days alive and out of the hospital over 90 days (35–38). However, a large number of patients and a population-based design can enhance our model and reduce potential confounding effects. In addition, some new therapeutic approaches such as immunotherapy and molecularly targeted therapies are also important variables (39, 40), but there is a lack of relevant information in the SEER database. Since our study is retrospective, the results should be treated with caution. Lastly, even though we used an external validation cohort to validate the model, further prospective studies are still needed to validate our conclusions.

## Conclusion

In short, this study used a large number of cases with demographic information, clinicopathological factors, and treatment characteristics to construct and validate a nomogram for predicting the OS of HGBC patients after RC. This nomogram can help clinicians make follow-up plans and subsequent treatment options to improve patient prognosis.

## Data availability statement

The original contributions presented in the study are included in the article/supplementary material. Further inquiries can be directed to the corresponding authors.

## Ethics statement

This study was approved by the Institutional Review Board of the First Affiliated Hospital of Nanchang University. All data from the SEER database were open access.

## Author contributions

TC proposed the research idea. YLi collected the data and wrote the manuscript. TC and YLi completed the statistical analysis. LC, YLu and BF reviewed the research framework. All authors contributed to the article and approved the submitted version.
